# Streptavidin interfacing as a general strategy to localize fluorescent membrane tension probes in cells†
†Electronic supplementary information (ESI) available: Detailed procedures and results for all reported experiments. See DOI: 10.1039/c8sc03620a


**DOI:** 10.1039/c8sc03620a

**Published:** 2018-10-17

**Authors:** Antoine Goujon, Karolína Straková, Naomi Sakai, Stefan Matile

**Affiliations:** a School of Chemistry and Biochemistry , National Centre of Competence in Research (NCCR) Chemical Biology , University of Geneva , Geneva , Switzerland . Email: stefan.matile@unige.ch ; http://www.unige.ch/sciences/chiorg/matile/

## Abstract

To image the mechanical properties of biological membranes, twisted push–pull mechanophores that respond to membrane tension by planarization in the ground state have been introduced recently. For their application in biological systems, these so-called fluorescent flippers will have to be localized to specific environments of cellular membranes. In this report, we explore streptavidin as a versatile connector between biotinylated flipper probes and biotinylated targets. Fluorescence spectroscopy and microscopy with LUVs and GUVs reveal the specific conditions needed for desthiobiotin-loaded streptavidin to deliver biotinylated flippers selectively to biotinylated membranes. Selectivity for biotinylated plasma membranes is also observed in HeLa cells, confirming the compatibility of this strategy with biological systems. Streptavidin interfacing does not affect the mechanosensitivity of the flipper probes, red shift in the excitation maximum and fluorescence lifetime increase with membrane order and tension, as demonstrated, *inter alia*, using FLIM.

## Abstract 2

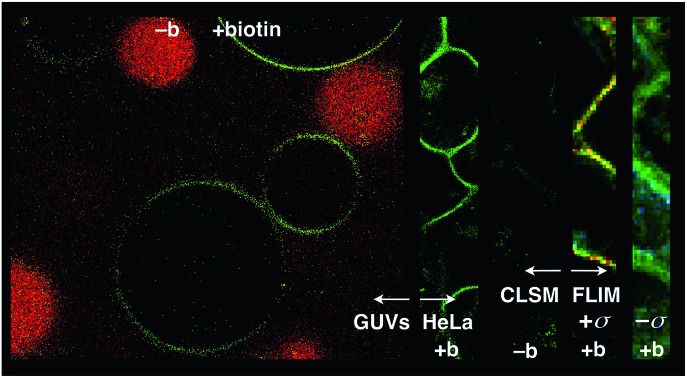
Site-specific labeling with biotinylated mechanophores is probed to address the next challenge toward the imaging of forces in cells.

## Introduction

The fluorescence imaging of membrane tension in living cells is one of the more demanding challenges in current biological research that awaits solutions from chemistry.^1^ The fundamental problem is that forces as such are not directly visualizable, neither in cells nor elsewhere. It is only their consequences that can be imaged. For membrane tension, the consequences are diverse, differ for different membranes, and are often unknown, which is also because reliable fluorescent probes for routine studies have not been available.

**Fig. 1:**
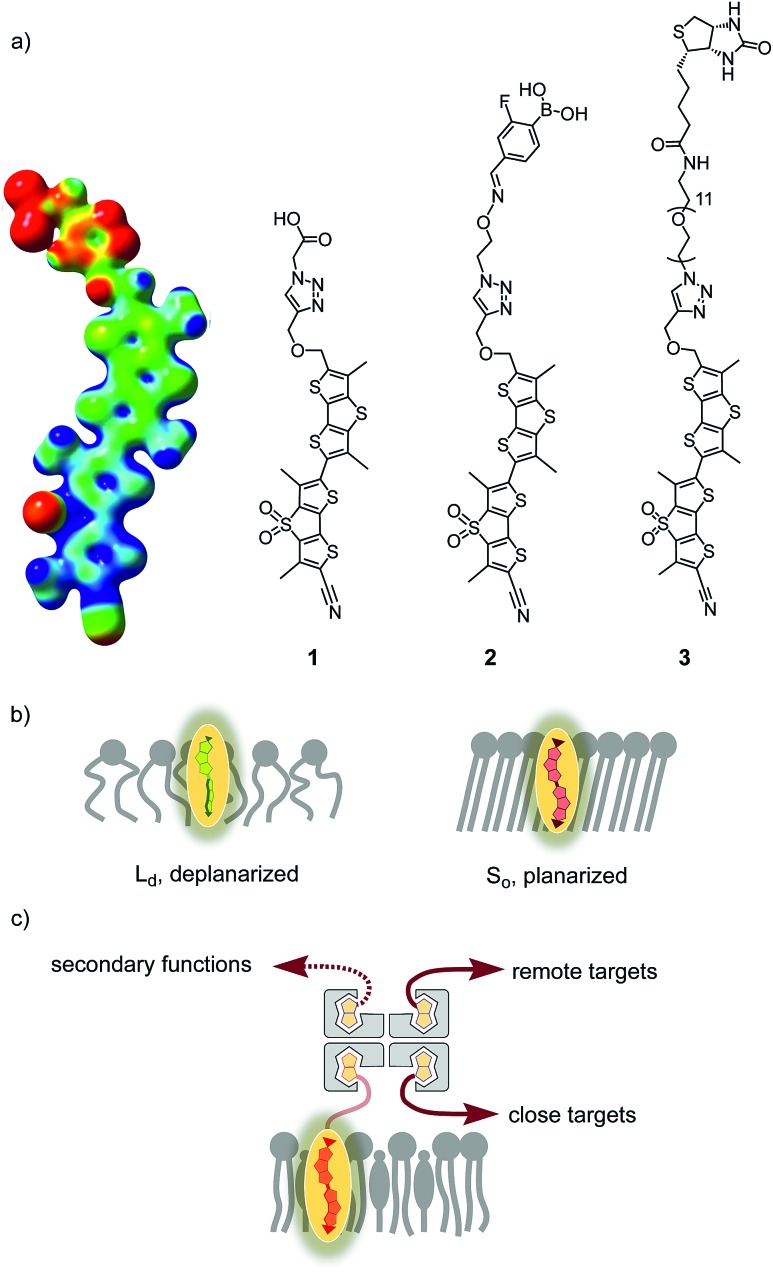
(a) Structures of original flipper **1** (with the MEP surface of the planarized conformer; red, electron rich; blue, electron poor), flipper **2** for ganglioside recognition, and flipper **3** introduced in this study; (b) schematic indication of increasing flipper planarization with increasing membrane order, from L_d_ to S_o_; and (c) the general concept of interfacing with close and remote targets through streptavidin. Relative orientations of ligands are arbitrary.

To image membrane tension in living cells, we have introduced the concept of planarizable push–pull probes,^2^ also referred to as “fluorescent flippers”.^3^ The current best mechanophore **1**, also called FliptR (fluorescence lipid tension reporter),^4^ is constructed around two dithienothiophene (DTT)^5^ “flippers” (Fig. 1).^6^ They excel with the high surface area needed for high mechanosensitivity and intense monomer fluorescence to keep shining when twisted out of conjugation. This deplanarization is achieved by “chalcogen-bond^7^ repulsion” between methyls and σ holes next to the twistable bond between the two DTT flippers. The polarization of the twisted mechanophores is achieved by using sulfone acceptors and sulfide donors as bridges in the two DTTs. The former are supported by a cyano acceptor, the latter by an essential thenyl ether, presumably for intramolecular chalcogen bonding. A triazole is used to prevent protonation and the resulting degradation of the thenyl ether.^6^ The terminal carboxylate is placed to produce amphiphiles that form soluble, non-fluorescent micelles in water.

In apolar solvents, the excitation maximum of flipper **1** is blue shifted. The increasing ground-state planarization of the push–pull probe with increasing order in lipid bilayer membranes shifts the excitation maximum to the red region (Fig. 1b).^3^ This red shift is accompanied by an increase in fluorescence intensity, *i.e.*, lifetime. These changes in the lifetime are well suited for fluorescence imaging of cells by FLIM (fluorescence lifetime imaging microscopy), a method that is attractive because the readout is independent of probe concentration.^4^,^6^ Little change in emission confirms that flippers **1** do not operate in the excited state like most other fluorescent membrane probes,^8^–^14^ which function with mechanisms such as solvatochromism,^8^ TICT (molecular rotors),^9^ ESIPT,^10^ PET,^11^ FRET,^12^ vibrational unbending,^13^ and so on. Rather than reporting off-equilibrium on kinetics, that is viscosity, planarizable push–pull probes thus report exclusively on mechanical confinement in space under equilibrium conditions in the ground state.^13^

Unlike previously proposed optical tension probes,^15^ FliptR **1** proved compatible with routine imaging of membrane tension in living cells.^4^ Increasing membrane tension in homogeneous model membranes, applied either by osmotic shock or micropipette aspiration, was found to result in a linearly decreasing fluorescence lifetime. This outcome is consistent with lipid decompression and flipper deplanarization as a response to membrane tension in homogeneous membranes. Increasing membrane tension in phase-separating model membranes as well as cells resulted in a linearly increasing fluorescence lifetime. This is consistent with lipid reorganization, that is the appearance and disappearance of membrane domains, as a dominant response to tension. Tension-induced lipid reorganization has been confirmed to occur in model membranes,^4^,^16^ and the FliptR probe has already been used to demonstrate the relevance of tension-induced lipid reorganization for biological function, that is signal transduction.^17^ Lipid reorganization as a dominant response to membrane tension suggested that other existing membrane probes could, in principle, image membrane tension as well. Considering the many parameters that influence fluorescence response,^4^ this remains to be confirmed probe by probe, particularly considering that flippers report in the ground state on sterics, whereas other probes report off-equilibrium in the excited state on kinetics, that is viscosity.^13^

Flipper **1** labels the outer membrane of cells, without strong preferences for different domains. For biological studies, however, it is essential to localize membrane tension probes to specific membrane environments. Preliminary results in this direction have been obtained using a boronic acid containing flipper, **2**, which was shown to partition better in ganglioside-enriched lipid domains of mixed-phase vesicles.^18^ This approach has potential to be extended for selective labeling of cellular organelles, such as mitochondria,^19^ ER, lysosomes and endosomes,^20^ through attachment of the well-established targeting units. On the other hand, selective tagging will be necessary to gain higher resolution insight into a particular protein. Compatibility of the Halo tag with molecular rotors has just been demonstrated,^21^ and SNAP tags^22^ and native ligand binding^23^ have been used to label the plasma membrane around surface receptors. In these pioneering studies on targeting, mechanophores were usually expected to report on organelle viscosity, and other probes were used just for labeling; all studied without explicit interest in lipid bilayer membranes, certainly not membrane tension.

**Fig. 2:**
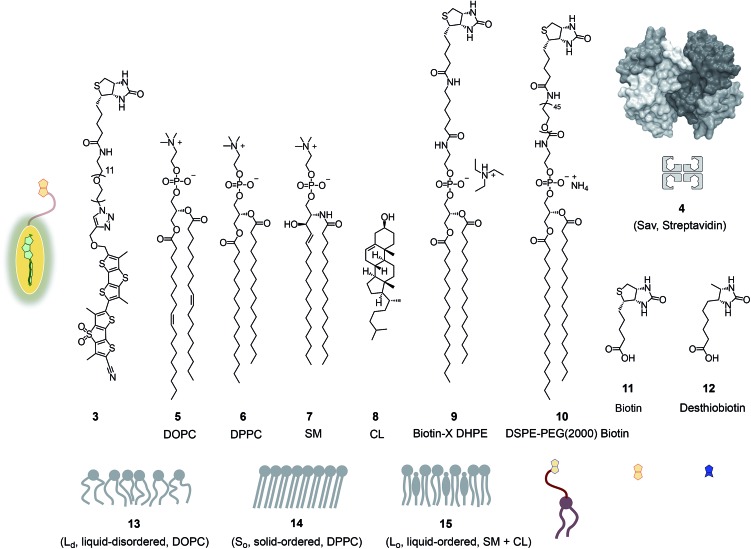
Structures, abbreviations, cartoons and numbering of molecules and molecular systems used in this study.

In this report, we explore the scope and limitations of streptavidin as a universal connector of tension probes with biotinylated targets (Fig. 1c and 2). Streptavidin–biotin interfacing is one of the best explored methods in biotechnology.^24^–^28^ The multivalency of the streptavidin tetramer provides unique versatility; examples extend from the combination of cellular uptake with fluorescent labeling, molecular recognition, self-assembly and catalysis^24^ to the construction of ordered multicomponent architectures on solid surfaces (Fig. 1c).^25^ Of particular importance for bioconjugation applications is the AviTag technology, which allows the attachment of biotin ligands at specific positions in proteins of free choice.^26^ In the following, we introduce biotinylated flipper probe **3** (Fig. 1a) and elaborate on the interfacing with streptavidin **4** to biotinylated lipids in large unilamellar vesicles (LUVs) and giant unilamellar vesicles (GUVs) of different order, and in cells.

## Results and discussion

Fig. 2 shows a summary of structures, abbreviations, cartoons and numbering of molecules and molecular systems **3–15** used in this study. The syntheses of flipper probes **1** and **2** have been reported.^6^,^18^ To prepare biotinylated flipper **3**, the CuAAC reaction was performed between the alkyne intermediate and an oligoethyleneglycol containing one azide and one amine terminus, and the resulting product was reacted with the activated NHS ester of biotin **11** (Scheme S1†).

### Biotinylated flippers

**Fig. 3:**
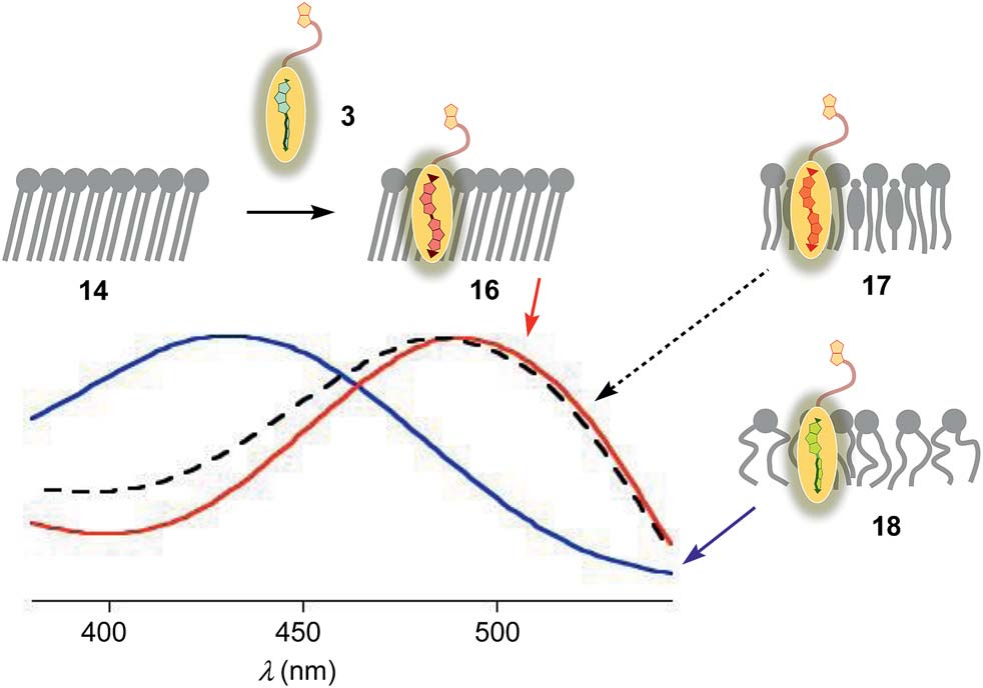
Normalized excitation spectra (*λ*_em_ = 570 nm) of flipper **3** (250 nM) in S_o_ (**16**, red), L_o_ (**17**, black) and L_d_ (**18**, blue) LUVs at a constant lipid concentration (75 μM) in 10 mM Tris, pH 7.4, 25 °C.

The properties of flipper **3** in lipid bilayer membranes were examined at 25 °C in LUVs of different composition: DOPC **5** (1,2-dioleoyl-*sn*-glycero-3-phosphocholine) for liquid-disordered (L_d_) membranes **13**, DPPC **6** (1,2-dipalmitoyl-*sn*-glycero-3-phosphocholine) for solid-ordered (S_o_) membranes **14**, and a mixture of SM **7** (sphingomyelin) and CL **8** (cholesterol) for liquid-ordered (L_o_) membranes **15** (Fig. 2). The probe was added to the vesicles (75 μM lipid) in Tris buffer, pH 7.4, 25 °C, to reach final concentrations of 1.0, 0.75, 0.5 and 0.25 μM. When added to S_o_ membranes **14**, mechanophore **3** gave a broad excitation peak with a maximum at *λ*_ex_ = 490 nm (Fig. 3, red, solid). This red shifted *λ*_ex_ demonstrated partitioning and planarization of the push–pull probe within the highly ordered membrane **16**. The excitation maximum obtained in L_o_ membranes **17** was nearly the same as in S_o_ membrane **16** (Fig. 3, black, dashed). In contrast, the excitation maximum obtained in L_d_ membranes **18** was clearly blue shifted at *λ*_ex_ = 430 nm (Fig. 3, blue, solid). This sensitivity toward membrane order was almost the same as with the original flipper probe **1**.^6^ The increasing planarization of the biotinylated flipper **3** with increasing membrane order fully confirmed its operational mechanosensitivity.

**Fig. 4:**
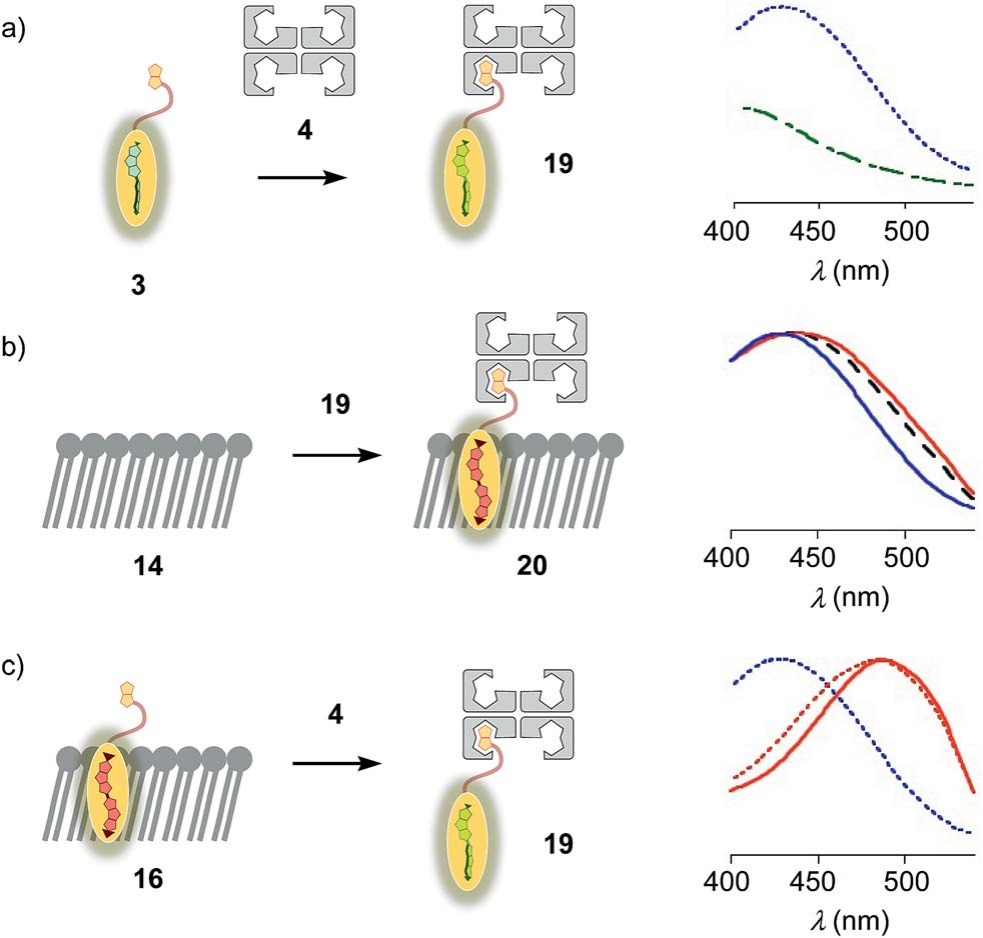
(a) Excitation spectra of flipper **3** before (dashed, green) and after the addition of one equivalent of **4** to yield **19** (dotted, blue). (b) Normalized excitation spectra recorded after the addition of **19** to membranes **13** (DOPC, solid, blue), **14** (DPPC, solid, red) and **15** (SM/CL, dashed, black), showing poor insertion of the probe into the membrane to yield complex **20** or equivalent. (c) Normalized excitation spectra of **16** (solid, red), **19** (dotted, blue) and **16** after addition of **4** (dotted, red), showing poor extraction of the probe from the membrane.

Flipper–streptavidin complex **19** was prepared by adding one equivalent of flipper **3** per wild-type streptavidin tetramer **4** in a buffer at pH 7.4, at room temperature (Fig. 4a). Compared to the very weak fluorescence of the biotinylated flipper **3** in buffer (Fig. 4a, green, dashed), the formation of complex **19** caused an increase in intensity and a red shift of the excitation maximum to *λ*_ex_ ∼ 430 nm (Fig. 4a, blue, dotted). The excitation spectra of flipper **3** bound to streptavidin **4** (*i.e.*, **19**) and L_d_ membranes (*i.e.*, **18**) were very similar. For the study of membrane-bound flippers, including cellular imaging, this similarity was irrelevant because the 4.8 times higher fluorescence intensity of flippers bound to L_d_ membranes (*i.e.*, **18**) made eventual background contributions from flipper–streptavidin complexes **19** in solution negligible (*vide infra*). Similar shifts at weaker intensity could suggest that in flipper–streptavidin complexes, the mechanophore interacts weakly with more hydrophobic domains on the protein surface^24^ to experience similar planarization but more rotational quenching compared to that in L_d_ membranes. In normalized spectra, eventual binding of **19** to L_d_ membranes **13** could thus not be detected from shifts of the excitation maxima. For the binding studies in LUVs described in the following, this overlap is irrelevant because the focus is on the more demanding and more informative S_o_ DPPC membranes **14**. The red shifted *λ*_ex_ = 490 nm of planarized mechanophores in these ordered membranes is readily detectable. Results with L_o_ SM/CL membranes **15** were often very similar to those in S_o_ DPPC **14**.

The addition of flipper–streptavidin complex **19** to biotin-free S_o_ membranes **14** caused only a small peak broadening toward longer wavelengths in the excitation spectrum (Fig. 4b, red, solid). Spectral deconvolution,^18^ assuming contributions from membrane bound **20** and the unbound **19** only, suggested that the yield of complex **20** with planarized flippers in S_o_ membranes is 16%, while the large majority of flippers bound to streptavidin in complex **19** remain in solution. Similarly poor partitioning could be observed with L_o_ membranes **15** (13%, Fig. 4b, black, dashed). Reverse addition of streptavidin **4** to flipper–membrane complex **16** did not change much the red shifted excitation maxima of planarized flippers in S_o_ membranes (Fig. 4c, red, dashed red). Whereas complexation with streptavidin in **19** thus hindered the insertion of flippers into ordered membranes, streptavidin **4** failed to extract flippers **3** from ordered membranes. These differences did not disappear with time; spectra measured after 15 and 30 min were unchanged. They suggested that the consequences of spatial confinement are important: binding to one partner hinders accessibility to the other. For flipper–streptavidin interactions, this conclusion was consistent with the red shift found for complex **19** compared to unbound flipper **3** (Fig. 4a).

### Biotinylated lipids

The schematic structures of all streptavidin complexes including **19** show the molar ratios of the components used. In reality, the multivalency of streptavidin complicates the situation.^26^–^28^ Without cooperativity effects, the complex stoichiometries reflecting the substrate ratio should dominate clearly.^27^ Conflicting reports suggest that cooperativity depends on the nature of the biotin ligand,^27^ and that, if desired, stoichiometries and structures of the complex can be controlled with mutants.^26^,^28^ It is generally accepted that the second biotin binds preferentially at the distant (≈3.5 nm) *trans* binding site rather than at the nearby (≈2.0 nm) *cis* site. Binding of the third and the fourth biotin ligands, inevitably at the *cis* positions of the first two ligands, could suffer from steric or charge repulsion. Anchoring of streptavidin on the bilayer surface requires divalent binding in the *cis* or *trans* orientation, and the remaining free sites can interact with more ligands.^25^ The availability of free binding sites could eventually be of interest to efficiently interface flipper probes with membrane proteins, either through biotinylated ligands of these receptors or strategically bioengineered biotin tags.^26^

**Fig. 5:**
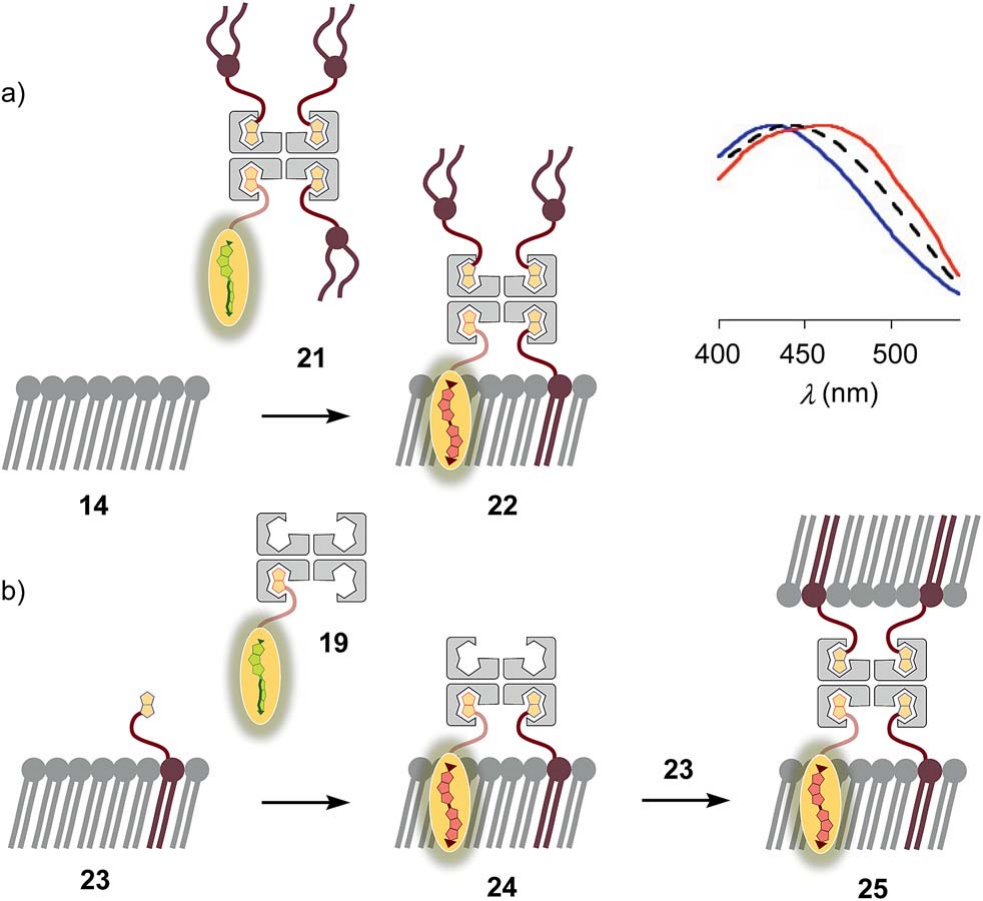
(a) Normalized excitation spectra recorded after the addition of complex **21** to membranes **13** (DOPC, blue), **15** (SM/CL, black) and **14** (DPPC, red; according to spectral deconvolution, formation of complex **22** occurred in 30%). (b) Fast vesicle precipitation resulted after the addition of complex **19** to biotinylated membranes **23**.

The biotinylated lipid **9** has been used previously to, for example, immobilize liposomes on streptavidin-coated surfaces, probe phosphoinositide–protein interactions, or assemble liposomes (Fig. 2).^29^ The addition of flipper–streptavidin–lipid complex **21** with, on average, one flipper **3** and three lipids **9** to S_o_ membranes **14** afforded complex **22** with planarized mechanophores in only 30%, according to spectral deconvolution (Fig. 5a, red, solid). Partitioning of complex **21** into L_o_ membranes **15** was even poorer (17%, Fig. 5a, black, dashed).

The complementary addition of flipper–streptavidin complex **19** to biotinylated S_o_ DPPC membranes **23** caused intense precipitation (5 mol% **9**, Fig. 5b). Dominant precipitation from complex **24** was consistent with the crosslinking of vesicles through streptavidin binding to biotins in different membranes (Fig. 1c) to form complex **25**.29*c*

**Fig. 6:**
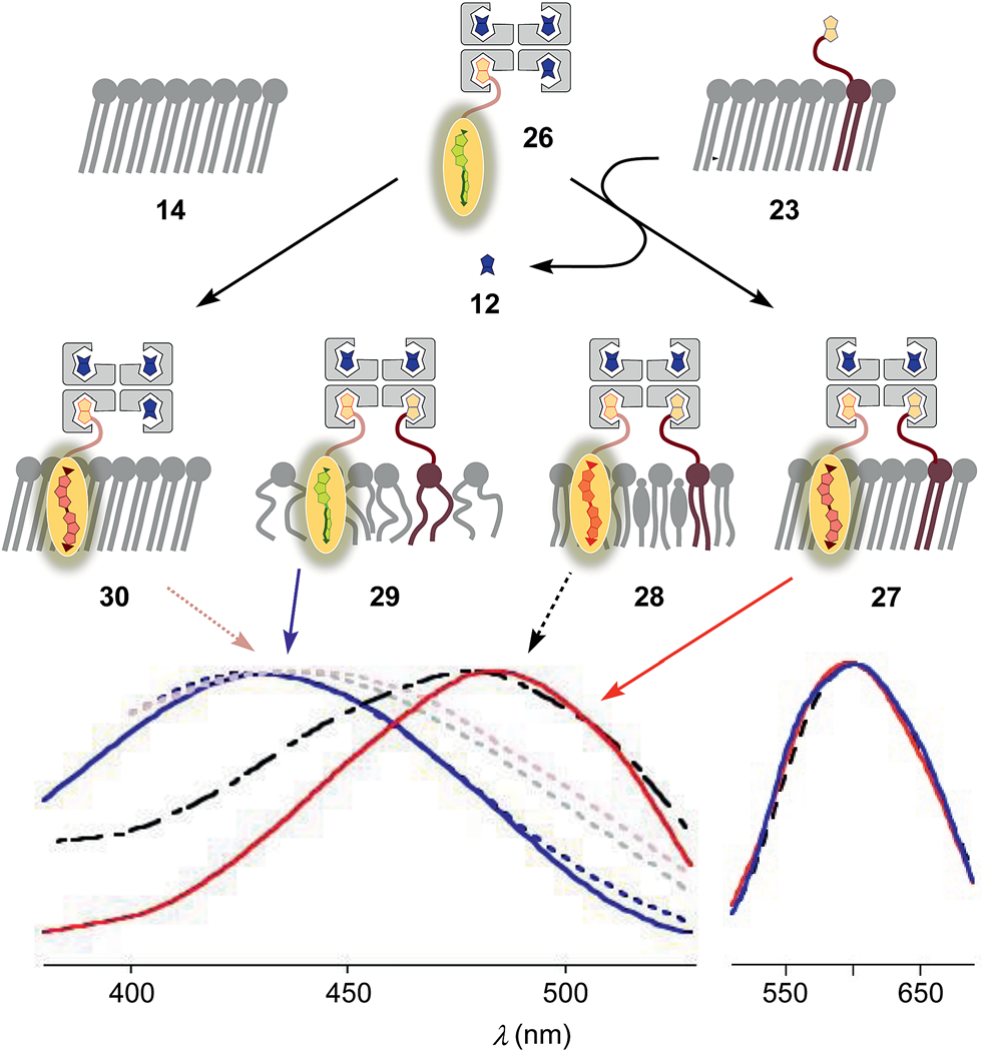
Normalized excitation (left) and emission spectra (right) recorded after the addition of complex **26** (250 nM) to L_d_, L_o_ and S_o_ (**23**) membranes with 5 mol% biotinylated lipid **9** to afford **29** (blue, solid), **28** (black, dashed) and **27** (red, solid), respectively. Control experiments show that addition of **26** to non-biotinylated L_d_ (blue, dotted), L_o_ (grey, dotted) and S_o_ (**14**, red, dotted) membranes results in poor binding (*e.g.*, **30**). 75 μM lipid, 10 mM Tris, pH 7.4, 25 °C.

To inhibit the formation of insoluble aggregates **25**, the free binding sites in flipper–streptavidin complex **19** were “protected” with desthiobiotin **12** (Fig. 2 and 6). Desthiobiotin **12** has a high affinity for the binding pocket of streptavidin **4**, but lower than that of biotin **11** itself (*K*_D_ (**11**) = 40 fM, *K*_D_ (**12**) = 500 fM).^30^ Upon addition of flipper–streptavidin–desthiobiotin complex **26** to biotinylated S_o_ membranes **23**, precipitation was not observed, even after fifteen minutes. The red shifted excitation maximum was consistent with flipper planarization in S_o_ membranes, that is the successful formation of the desired interfaced architectures **27** (Fig. 6, red, solid). Similar flipper planarization was observed for the corresponding complex **28** in SM/CL L_o_ vesicles (Fig. 6, black, dashed), whereas the spectral signature of the interfaced complex **29** was consistent with that of the twisted flippers in L_d_ vesicles (Fig. 6, blue, solid).

Control experiments with non-biotinylated S_o_ membranes **14** gave insignificant red shifts upon addition of flipper–streptavidin–desthiobiotin complexes **26**, confirming that the formation of complex **30** is negligible (Fig. 6, red, dotted). Control experiments with non-biotinylated L_o_ and L_d_ membranes gave similarly poor partitioning (Fig. 6, grey and blue, dotted). These consistent trends confirmed that desthiobiotins in complex **26** are efficiently displaced by the biotinylated lipids in membranes **23** and equivalent, leading to the insertion of the mechanosensitive probes into the membrane and formation of the correctly interfaced architectures **27–29**.

The emission maxima of the interfaced flipper complexes **27–29** were almost identical (Fig. 6). This was as with non-interfaced flippers^3^,^6^ and confirmed that mechanosensitivity originates from planarization in the ground state in response to sterics.

**Fig. 7:**
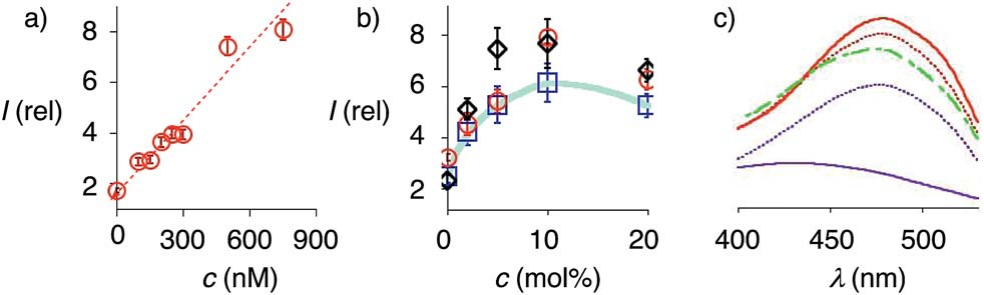
(a) Dependence of fluorescence intensity on the concentration of complex **26** after addition to L_o_ SM/CL LUVs containing 5 mol% **9**. (b) Relative fluorescence intensity of complex **26** (250 nM) after addition to DOPC (blue squares), DPPC (red circles) and SM/CL (black diamonds) LUVs containing 0 to 20 mol% **9**. (c) Excitation spectra of complex **26** recorded after addition to SM/CL LUVs with 0 (purple, solid), 2 (purple, dotted), 5 (red, dotted), 10 (red, solid) and 20 mol% **9** (green, dashed). Shown are the mean values ± standard errors from three independent experiments.

At a constant biotin level in L_o_ membranes, the dependence of fluorescence intensity on flipper concentration was roughly linear up to at least 800 nM (Fig. 7a). Similar observations were made for L_d_ membranes (Fig. S1a†), while progressive saturation was monitored at higher concentrations in S_o_ membranes (Fig. S2a†). With ≈2 μM biotin available on the LUV surfaces, these results were consistent with the need for less than two equivalents of biotinylated lipids to anchor the streptavidin complex. The absence of evident saturation at submicromolar concentrations further supported that the interfaced flipper complexes **27** and equivalent operate as monomers and do not aggregate under these conditions.

At constant flipper concentration, the dependence of the fluorescence intensity, *i.e.*, the formation of the interfaced complexes **27–29**, on the concentration of biotinylated lipids **9** was bell-shaped for all membranes tested, with a maximum around 10 mol% (Fig. 7b, c, S1b and S2b†). Several explanations for this saturation and ultimately decrease at higher mole fractions were conceivable. The decreasing intensity coincided with peak broadening at a shorter wavelength, indicating hindered flipper partitioning and/or planarization due to the disturbed organization of these over-biotinylated membranes (Fig. 7c, green).

**Fig. 8:**
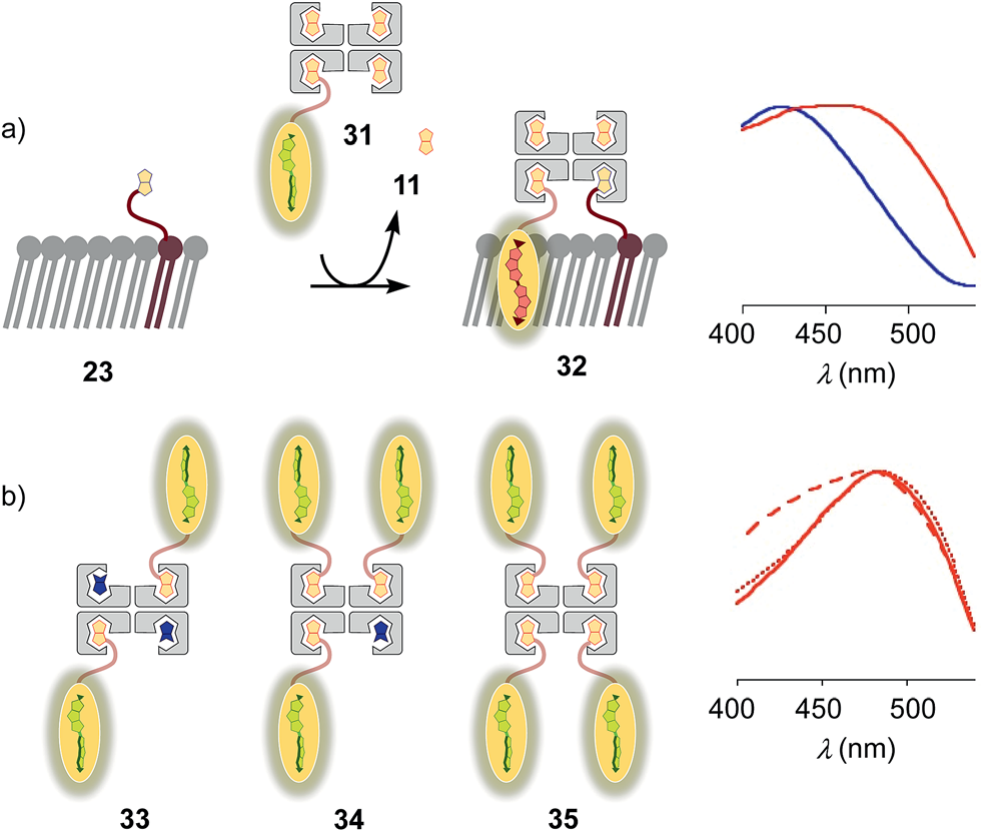
(a) Normalized excitation spectra of complex **31** after addition to DOPC (blue) and DPPC (red) LUVs prepared with 5 mol% **9**. (b) Normalized excitation spectra of complex **26** (solid), **33** (dashed) and **34** (dotted) recorded after addition to **23** (*i.e.*, DPPC LUVs prepared with 5 mol% **9**). Recorded in LUVs at constant lipid concentration in 10 mM Tris, pH 7.4, 25 °C, with a concentration of 1 μM of probe **3**.

Substitution of desthiobiotin **12** in complex **26** by biotin **11** in flipper–streptavidin–biotin complex **31** hindered efficient flipper interfacing (Fig. 8a). The addition of complex **31** to biotinylated S_o_ membranes **23** gave a broad excitation peak with a maximum at 480 nm but a shoulder extending to 430 nm that is characteristic of incomplete flipper planarization. Spectra measured after 15 min were unchanged. Spectral deconvolution suggested that only 30% of complex **32** was formed with vesicles that were biotinylated with 5 mol% of lipid **9**. This incomplete formation of complex **32** supported that the displacement of biotin or the biotinylated flipper is slow, and rapid desthiobiotin–biotin exchange is essential for correct and efficient interfacing.

With increasing flipper content from 2 : 2 complex **33** to 3 : 1 complex **34** and 4 : 0 complex **35**, the spectral signature of the target complex **27** did not improve with regard to flipper planarization, *i.e.*, red shift (Fig. 8b). Spectral deconvolution revealed 77% insertion for **33** and 93% for **34** compared to **26**. Red shift recovery from **33** to **34** was likely due to the displacement of a flipper **3** upon binding with **9**, which then directly partitions into the membrane and increases the proportion of the planarized probe in the spectra with contributions from non-interfaced flippers **16** (Fig. 3). Further increasing flipper content in the pure 4 : 0 flipper–streptavidin complex **35** caused intense and instantaneous precipitation, possibly due to the partitioning of *cis* and *trans* flippers into different vesicles (as outlined for lipids in **25**, Fig. 5).

**Fig. 9:**
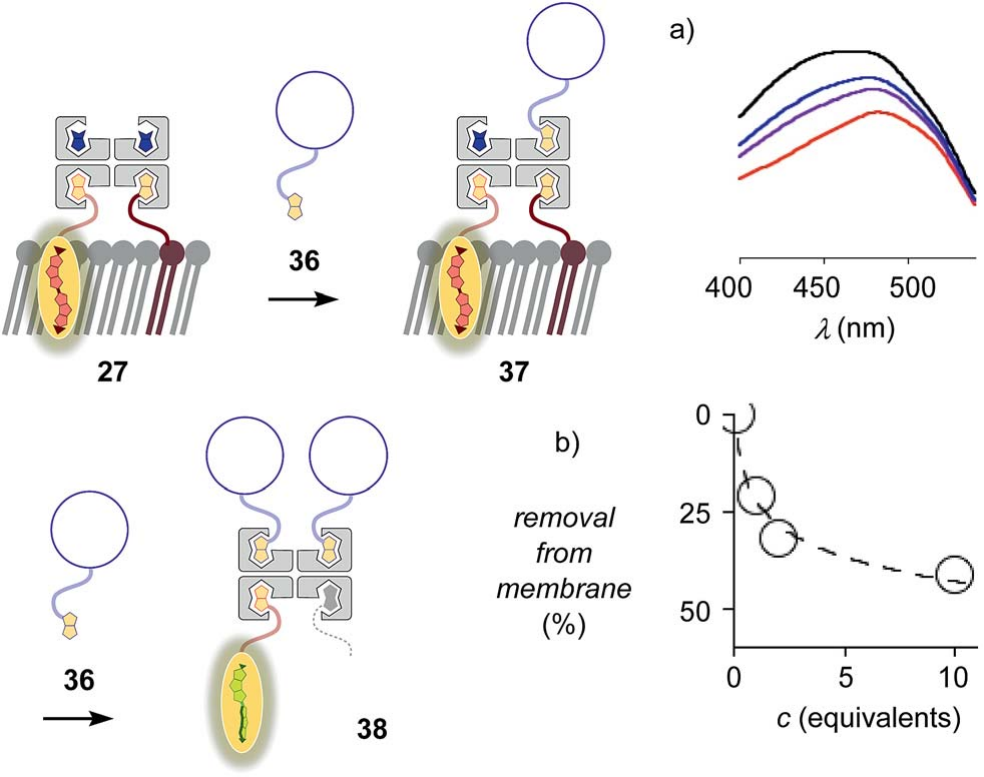
(a) Excitation spectra of complex **27** after the addition of 0 (red), 1 (purple), 2 (blue) and 10 (black) equivalents of biotinylated insulin **36**. (b) Flipper removal from the membrane with increasing concentration of insulin, obtained from deconvolution of spectra in (a).

The addition of biotinylated insulin **36** to the operational, correctly interfaced target complex **27** caused a gradual broadening of the excitation maxima of the planarized flippers toward the blue region (Fig. 9a). This result was consistent with the formation of first complex **37** with four different ligands bound to the tetravalent streptavidin, followed by flipper extraction from the membrane with complex **38** or similar, with two insulins and maybe also lipid **9** displaced by another insulin **36**. Spectral deconvolution gave 41% flipper removal in the presence of ten equivalents of insulin **36** (Fig. 9b). This reluctant flipper removal from S_o_ membranes implied the formation of non-interfaced flipper **16***via* displacement of biotinylated flipper **3** in complex **37** by biotinylated insulin **36**.

### Fluorescence imaging in GUVs

**Fig. 10:**
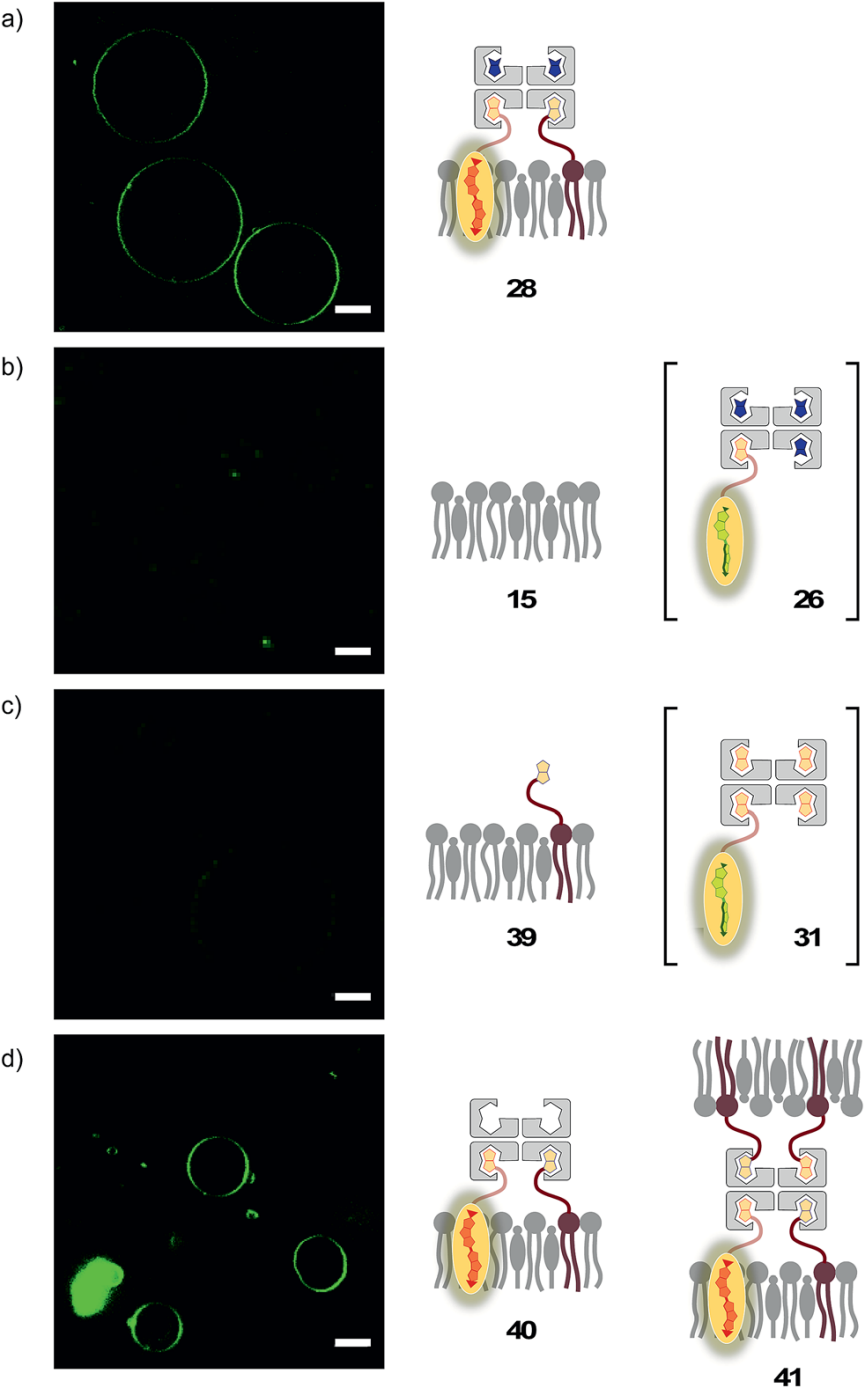
CLSM images of complex **26** added to (a) biotinylated (yielding **28**) and (b) non-biotinylated L_o_ SM/CL GUVs, and (c) complex **31** and (d) complex **19** added to biotinylated L_o_ SM/CL GUVs. Pictures were taken 2 min after the addition of complexes, 500 nM **3**, *λ*_ex_ = 488 nm, *λ*_em_ = 600 ± 50 nm, 30% laser power, scale bar 10 μm.

The lessons learned in LUVs were applied to imaging flipper interfacing in GUVs. For convenience only, the studies were carried out mostly in L_o_ SM/CL membranes **15** (Fig. 2). Confocal laser scanning microscopy (CLSM) images of biotinylated L_o_ membranes **39** (5 mol% **9**) after addition of flipper–streptavidin complex **26** with exchangeable desthiobiotin **12** in the extra binding sites showed cleanly labeled GUVs without any precipitation *i.e.* the desired target complex **28** (Fig. 10a). The same flipper–streptavidin–desthiobiotin complex **26** failed to label L_o_ SM/CL membranes **15** without biotin on their surface (Fig. 10b). Moreover, the flipper–streptavidin complex **31** with poorly exchangeable biotin **11** rather than the readily substituted desthiobiotin **12** failed to label biotinylated L_o_ SM/CL membranes **39** (Fig. 10c). Finally, the addition of flipper–streptavidin complex **19** with neither desthiobiotin **12** nor biotin **11** in the extra binding sites produced labeled GUVs together with small and also very large precipitates, *i.e.*, architectures **40** and **41** (Fig. 10d).

**Fig. 11:**
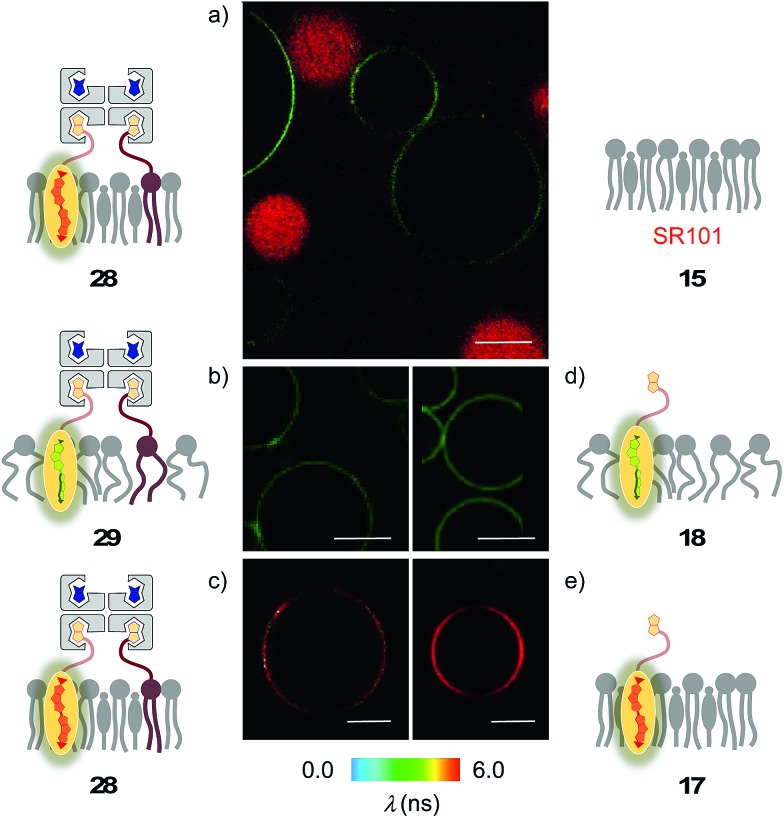
(a) CLSM images of complex **26** added to a mixture of SM/CL/**9** (5%) GUVs (yielding **28**, green) and SR101-loaded SM/CL GUVs (red). (b–e) FLIM images of complex **26** added to (b) DOPC/**9** (5%) GUVs (yielding **29**) and (c) SM/CL/**9** (5%) GUVs (yielding **28**), and of flipper **3** added to (d) DOPC GUVs (yielding **18**) and (e) SM/CL GUVs (yielding **17**). Pictures were taken after 2 min, 500 nM **3**, *λ*_ex_ = 488 nm, *λ*_em_ = 600 ± 50 nm, 30% laser power, scale bar 10 μm.

Biotin-free L_o_ SM/CL GUVs **15** loaded with sulforhodamine 101 (SR101) were imaged together with biotinylated L_o_ SM/CL GUVs **39** after the addition of the flipper–streptavidin–desthiobiotin complex **26**. Consistent with the formation of the target complex **28**, the membrane of biotinylated GUVs **39** could be clearly observed (in green), whereas the non-biotinylated but SR101-loaded GUVs **15** (in red) did not show fluorescently labeled membranes (Fig. 11a). This series of CLSM images confirmed that the correctly interfaced complex **28** is accessible exclusively by the addition of the flipper–streptavidin–desthiobiotin complex **26** to biotinylated membranes **39** (Fig. 10a and 11a) because the presence of desthiobiotin is essential (Fig. 10c and d) and, most importantly, non-biotinylated membranes are not labeled (Fig. 10b and 11a). The results with GUVs were in full agreement with the spectroscopic analysis in LUVs (Fig. 4b, 5b, 6, and 8a). GUV imaging thus validated the addition of the flipper–streptavidin–desthiobiotin complex **26** to biotinylated targets as the winning strategy for operational interfacing.

FLIM was performed after the addition of the flipper–streptavidin complex **26** to biotinylated L_o_ SM/CL GUVs **39** (Fig. 11c). The fluorescence lifetime *τ*^L_o_^ = 5.7 ns obtained for the interfaced L_o_ complex **28** was in the range between *τ*^L_o_^ = 6.1 ns measured after the addition of flipper **3** to L_o_ SM/CL GUVs, *i.e.*, complex **17** (Fig. 11e), and *τ*^L_o_^ = 5.8 ns reported^6^ for the original flipper **1** in L_o_ membranes. Consistent with the lifetime of the original flipper **1** in L_d_ membranes, the fluorescence lifetimes obtained with flipper **3** in L_d_ DOPC GUVs, *i.e.*, complex **18**, were much shorter (*τ*^L_d_^ = 3.8 ns, Fig. 11d). FLIM images of flipper–streptavidin–desthiobiotin complex **26** added to biotinylated L_d_ DOPC GUVs, *i.e.*, the interfaced L_d_ complex **29**, gave the same lifetime (*τ*^L_d_^ = 3.8 ns, Fig. 11b). These trends confirmed that the fluorescence property of planarized flippers in adequately interfaced L_o_ architectures **28** and deplanarized flippers in L_d_ architectures **29** is similar to that of flippers in the absence of streptavidin. In other words, streptavidin interfacing did not disturb the operation of flipper probes and is thus compatible with FLIM imaging of rationally localized membrane tension in cells.

### Fluorescence imaging in cells

Cell surface biotinylation was achieved by growing HeLa Kyoto cells for three days in the presence of DSPE-PEG(2000) biotin **10** (Fig. 2). This lipid has been used routinely to, for example, immobilize GUVs on the surface, quantify the uptake of viruses in cells, or study the partitioning of synthetic lipids in mixed phase GUVs.^4^,^31^ The biotinylated lipid **9** used in vesicles could not be used in cells because it was not soluble enough in solvents miscible with water (methanol, DMSO, *etc.*) to be added to the cellular growth medium.

**Fig. 12:**
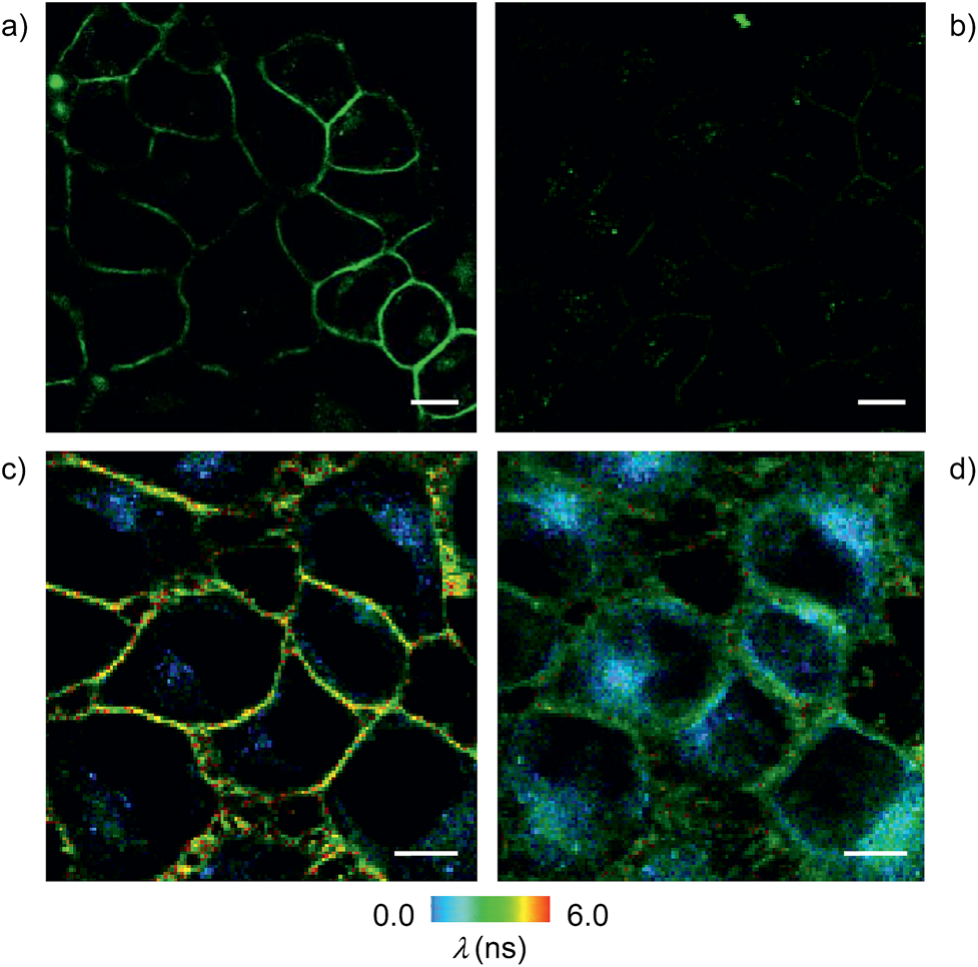
CLSM images of HeLa Kyoto cells with (a) and without (b) pre-incubation with biotinylated lipid **10** (25 μg mL^–1^) for 3 days, followed by washing and incubation with **26** (2 μM, 5 min; scale bar: 10 μm). FLIM images of HeLa cells incubated with **10** (25 μg mL^–1^) for 3 days followed by washing and incubation with **26** (2 μM, 5 min) under (c) isoosmotic and (d) hyperosmotic conditions.

The addition of the operational flipper–streptavidin–desthiobiotin complex **26** to the biotinylated HeLa cells selectively stained the plasma membrane with little background signal (Fig. 12a). In contrast, the addition of complex **26** to HeLa cells without biotinylation did not result in any significant fluorescence (Fig. 12b). FLIM experiments with interfaced flipper **3** in the biotinylated plasma membrane of HeLa cells gave a fluorescence lifetime of *τ* = 5.5 ns (Fig. 12c). This lifetime was similar to the one measured for the original flipper **1** in the plasma membrane of HeLa cells.^4^ Under hyperosmotic conditions, the lifetime of the interfaced flipper **3** in the plasma membrane of HeLa cells decreased significantly to *τ* = 4.95 ns (Fig. 12d). This response to the reduction of membrane tension was as with the original flipper **1**.^4^ The decrease of flipper lifetime with tension has been proposed to originate from tension-induced lipid reorganization dominated by the disappearance of highly ordered domains with long-lived, strongly emitting planarized flipper probes.

In sharp contrast to the original flipper probe **1**, hyperosmotic shock not only reduced the lifetime of interfaced flippers in the plasma membrane but also caused rapid partial internalization and the appearance of punctate spots with a very short lifetime: *τ* = 2.8 ns. Although explanations on their origin remain to be found, the different responses to membrane tension observed with the original flipper **1** and interfaced flipper **3** beautifully forecast the specific information that will become available with the introduction of interfacing strategies to rationally localize flipper mechanophores within cells.

## Conclusions

With fluorescent probes for the routine imaging of membrane tension in cells in hand,^4^ the next milestone will be the development of a universal interfacing strategy to measure membrane tension at any place in any living cell. This report suggests that streptavidin interfacing can meet this important challenge. The key to success was to protect extra binding sites in the tetrameric interface with exchangeable desthiobiotin (Fig. 6). Streptavidin complexes with a biotinylated flipper and desthiobiotin exchangers are shown to specifically label membranes that contain biotinylated lipids. As shown by fluorescence spectroscopy and FLIM, the probe retains its mechanosensitive properties even if it is part of such large supramolecular architectures, and can reveal unique characteristics of the target. Preliminary results for staining biotinylated plasma membranes are promising (Fig. 12). Compatibility with further interfacing to membrane proteins, either through biotinylated ligands or engineered AviTags,^26^ is demonstrated.

The potential identified in this paper will have to be validated in studies on real biological problems and compared to other approaches such as Halo tags,^21^ SNAP tags^22^ or IEDDA ligation with artificial amino acids in engineered proteins.^32^ The unique versatility of a connector with four similar but non-identical binding sites is advantageous and disadvantageous at the same time. One disadvantage is the presence of mixtures of complexes with different stoichiometries. However, our findings suggest that this unsatisfactory heterogeneity has surprisingly little relevance when it comes to the specific labeling of biotinylated membranes in practice (Fig. 11 and 12). A general advantage of tetravalent interfacing is access to multiple functionalities. For example, membrane interfacing can be coupled with protein interfacing (Fig. 9), or with cellular delivery vehicles.^24^

## Experimental section

See ESI.†

## Conflicts of interest

There are no conflicts to declare.

## Supplementary Material

Supplementary information
