# Supplementary material for: Streptavidin interfacing as a general strategy to localize fluorescent membrane tension probes in cells
Source: Chem Sci. 2018 Oct 17;10(1):310–9. doi: 10.1039/c8sc03620a (PMC6333237; doi:10.1039/c8sc03620a)
Supplement: Supplementary file 1 [file SC-010-C8SC03620A-s001.pdf]

Electronic Supplementary Material (ESI) for Chemical Science.
This journal is © The Royal Society of Chemistry 2018

Supporting Information

Streptavidin interfacing as a general strategy to localize fluorescent membrane tension probes in cells

Antoine Goujon, Karolína Straková, Naomi Sakai and Stefan Matile*

Department of Organic Chemistry, University of Geneva, Quai Ernest Ansermet 30, CH-1211 Geneva 4, Switzerland, e-mail: stefan.matile@unige.ch

Table of contents

1. Materials and methods S2
2. Synthesis S3
3. Streptavidin complexes S4
4. Preparation of LUVs S4
5. Fluorescence measurements in LUVs S6
6. Preparation of GUVs S7
7. Studies in GUVs S8
8. Studies in HeLa cells S8
9. NMR spectra S9
10. Supporting references S12

1. Materials and methods

As in ref. S1. Briefly, reagents for synthesis were purchased from Fluka, Sigma-Aldrich, TCI and Across. Salts of the best grade available from Fluka or Sigma-Aldrich were used as received. Egg sphingomyelin (SM), cholesterol (CL), 1,2-dioleoyl-*sn*-glycero-3-phosphocholine (DOPC), 1,2-dipalmitoyl-*sn*-glycero-3-phosphocholine (DPPC), DSPE-PEG(2000) biotin and mini-extruder were purchased from Avanti Polar Lipids. Wild-type streptavidin was a kind gift from Prof. T. R. Ward (University of Basel) and was used as received. Biotin-X DHPE was purchased from Thermofisher. Column chromatography was carried out on silica gel 60 (SiliaFlash P60, 40-63 μm). Analytical (TLC) and preparative thin layer chromatography (PTLC) were performed on silica gel 60 (Merck, 0.2 mm) and silica gel GF (SiliCycle, 1 mm), respectively. Reverse phase flash chromatography was performed on a Biotage® Isolera Spektra using pre-packed Biotage® SNAP Ultra C_{18} cartridges. Fluorescence measurements were performed using a FluoroMax-4 spectrofluorometer (Horiba Scientific) equipped with a stirrer and a temperature controller. Fluorescence spectra were corrected using correction factors supplied by the manufacturer. LCMS were recorded using a Thermo Scientific Accela HPLC equipped with a Thermo C_{18} Hypersil GOLD column (50 × 2.1 mm, 1.9 μm particles size) coupled with a LCQ Fleet three-dimensional ion trap mass spectrometer (ESI, Thermo Scientific) with a linear elution gradient from 70% H_2O / 30% CH_3CN + 0.1% TFA to 10% H_2O / 90% CH_3CN + 0.1% TFA in 4.0 minutes at a flow rate of 0.75 mL/min. IR spectra were recorded on a Perkin Elmer Spectrum 100 FT-IR spectrometer (ATR, Golden Gate) and are reported as wavenumbers υ in cm^{-1} with band intensities indicated as s (strong), m (medium), w (weak), br (broad). ^{1}H and ^{13}C spectra were recorded (as indicated) either on a Bruker 400 MHz or 500 MHz spectrometer and are reported as chemical shifts (δ) in ppm relative to TMS ($\delta = 0$). Spin multiplicities are reported as a singlet (s), doublet (d), triplet (t) and quartet (q) with coupling constants (J) given in Hz, or multiplet (m). Broad peaks are marked as br. ^{1}H and ^{13}C resonances were assigned with the aid of additional information from 1D and 2D NMR spectra (H,H-NOESY, H,H-COSY, DEPT 135, HSQC and HMBC). ESI-MS for the characterization of new compounds was performed on an ESI API 150EX and are reported as mass-per-charge ratio *m/z* (intensity in %, [assignment]). HR ESI-MS for the characterization of new compounds were performed on a QSTAR Pulsar (AB/MDS Sciex) and are reported as mass-per-charge ratio *m*/*z* calculated and observed. The confocal laser scanning microscopy images of the GUVs and HeLa cells were taken with a Leica SP5 STED. FLIM was performed on a Nikon Eclipse Ti A1R microscope upgraded with a FLIM kit from PicoQuant, equipped with a laser 485, PicoQuant, LDH-D-C-485 at 20 MHz.

Abbreviations. CL: Cholesterol; CLSM: Confocal laser scanning microscopy; DMEM: Dulbecco's modified eagle medium; DMF: Dimethylformamide; DOPC: Dioleyl-*sn*-glycero-3-phosphocholine; DPPC: Dipalmitoyl-*sn*-glycero-3-phosphocholine; FLIM: Fluorescence lifetime imaging microscopy; GUVs: Giant unilamellar vesicles; L_d: Liquid disordered; L_o: Liquid ordered; LUVs: Large unilamellar vesicles; NHS: N-hydroxysuccinimide; PBS: Phosphate-buffered saline; rt:

Room temperature, Sav: Streptavidin; S_o: Solid ordered; SM: Egg sphingomyelin; TBTA: Tris((1-benzyl-1H-1,2,3-triazol4-yl)methyl)amine; THF: Tetrahydrofuran; Tris: Tris(hydroxymethyl)aminomethane.

2. Synthesis

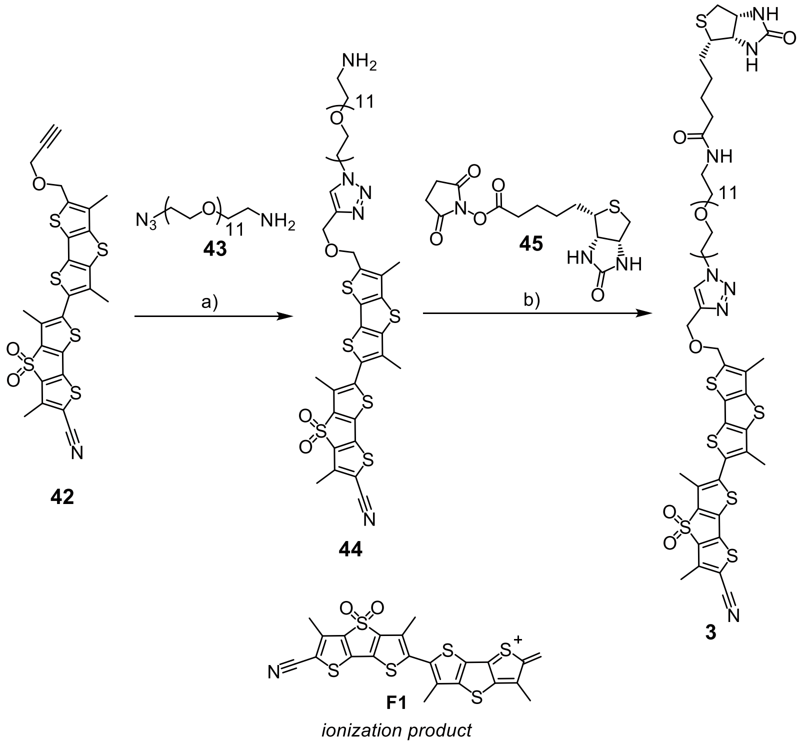

-

Scheme S1. (a) $CuSO_4.5H_2O$, Na-ascorbate, TBTA, CH_2Cl_2, H_2O, rt, 2 h; (b) triethylamine, DMF, THF, rt, 1 h.

Compound 42 was synthesized and purified according to procedures described in ref. S1.

Compound 43 was purchased from Iris Biotech and used as received.

Compound 44. Compound **42** (40 mg, 0.070 mmol), $CuSO_4.5H_2O$ (18 mg, 0.070 mmol), Na-ascorbate (14 mg, 0.070 mmol), TBTA (37 mg, 0.070 mmol) and compound **43** (48 mg, 0.084 mmol) were dissolved in a dichloromethane (2.6 mL) and water (0.6 mL) mixture. The solution was vigorously stirred for 2 h at rt, then water was added (10 mL), the mixture extracted with dichloromethane (2 x 10 mL), the organic phase was dried with Na_2SO_4, filtered and evaporated under reduced pressure. The crude red residue was dissolved in DMSO (2 mL) and purified on reverse phase flash chromatography (Biotage® SNAP Ultra C_{18} 12 g, CH_3CN / H_2O 20 – 100% gradient with 0.1% TFA) to yield **44** as a gummy red solid (72 mg, 90%). IR (neat): 3521 (w), 2916 (s), 2880 (s), 1668 (w), 1314 (m), 1136 (s), 1099 (s); ^{1}H NMR (400 MHz, $CDCl_3$): 7.79 (s, 1H), 4.80, (s, 2H) 4.73 (s, 2H), 4.56 (t, ^{3}J = 5.0 Hz, 2H), 3.88 (t, ^{3}J = 5.0 Hz, 2H), 3.72-3.68 (m, 44H), 3.05 (br s, 2H), 2.61 (s, 3H), 2.42 (s, 3H), 2.36 (s, 3H), 2.35 (s, 3H); ^{13}C NMR (101 MHz, $CDCl_3$): 145.4 (C), 144.7 (C), 143.8 (C), 143.2 (C), 142.4 (C), 141.4

(C), 140.3 (C), 138.3 (C), 136.1 (C), 132.6 (C), 132.5 (C), 132.2 (C), 131.4 (C), 129.4 (C), 128.9 (C), 125.6 (C), 124.1 (CH), 112.6 (C), 109.0 (C), 70.7 (7 CH_2), 70.6 (2 CH_2), 70.6 (2 CH_2), 70.6 (CH_2), 70.6 (CH_2), 70.5 (CH_2), 70.4 (CH_2), 70.4 (CH_2), 70.4 (2 CH_2), 70.3 (CH_2), 70.3 (CH_2), 70.3 (CH_2), 69.6 (CH_2), 65.4 (CH_2), 63.5 (CH_2), 50.4 (CH_2), 14.2 (CH_3), 13.3 (CH_3), 13.0 (CH_3), 12.6 (CH_3); MS (ESI+): 1142 (80, $[M+H]^+$), 516 (20, [**F1**]$^+$).

Compound 45 was synthesized and purified according to procedures described in ref. S2.

Compound 3. Compound **44** (60 mg, 0.053 mmol) was dissolved in THF (6 mL) and DMF (200 µL) along with triethylamine (36 µL, 0.27 mmol). NHS-ester **45** (18 mg, 0.053 mmol) was added in one portion and the reaction was stirred for 1 h at room temperature. The solvents were removed under reduced pressure to yield a crude red oil, which was dissolved in DMSO (2 mL) and purified on reverse phase flash chromatography (Biotage® SNAP Ultra C_{18} 12 g, CH_3CN / H_2O 20 – 100% gradient with 0.1% TFA) to yield **3** as a red oily film (49 mg, 69%). IR (neat): 3315 (w), 2921 (s), 2858 (s), 2211 (w), 1701 (s), 1540 (w), 1456 (m), 1352 (w), 1313 (m), 1142 (s), 1101 (s), 947 (w), 845 (w); ^{1}H NMR (400 MHz, DMSO-d_6): 8.11 (s, 1H), 7.80 (s, 1H), 6.39 (s, 1H), 6.33 (s, 1H), 4.78 (s, 2H), 4.63 (s, 2H), 4.53 (t, ^3J = 5.2 Hz, 2H), 4.32-4.28 (m, 1H), 4.14-4.09 (m, 1H), 3.81 (t, ^3J = 5.2 Hz, 2H), 3.52-3.47 (m, 40H), 3.38 (t, ^3J = 5.9 Hz, 2H), 3.17 (q, ^3J = 5.9 Hz, 2H), 3.11-3.05 (m, 1H), 2.81 (dd, ^2J = 12.4 Hz, ^3J = 5.1 Hz, 1H), 2.57 (d, ^2J = 12.4 Hz, 1H), 2.48 (s, 3H), 2.35 (s, 3H), 2.33 (s, 3H), 2.32 (s, 3H), 2.05 (t, ^3J = 7.4 Hz, 2H), 1.66-1.56 (m, 1H), 1.53-1.40 (m, 3H), 1.34-1.24 (m, 2H); ^{13}C NMR (101 MHz, DMSO-d_6): 172.1 (C), 162.7 (C), 143.9 (C), 143.5 (C), 142.2 (C), 142.2 (C), 141.7 (C), 140.7 (C), 139.8 (C), 138.0 (C), 136.9 (C), 133.2 (C), 131.4 (C), 130.5 (C), 130.2 (C), 128.5 (C), 127.9 (C), 125.9 (C), 124.5 (CH), 113.0 (C), 109.8 (C), 69.8 (18 CH_2), 69.6 (CH_2), 69.6 (CH_2), 69.2 (CH_2), 68.7 (CH_2), 64.5 (CH_2), 62.7 (CH_2), 61.0 (CH), 59.2 (CH), 55.4 (CH), 49.4 (CH_2), 39.58 (CH_2), 38.5 (CH_2), 35.1 (CH_2), 28.2 (CH_2), 28.0 (CH_2), 25.3 (CH_2), 13.8 (CH_3), 12.6 (CH_3), 12.5 (CH_3), 12.0 (CH_3); HRMS (ESI+) calcd. For $C_{59}H_{81}N_{16}O_{16}S_7$: 1368.3858 ($[M+H]^+$), found: 1368.3890.

3. Streptavidin complexes

To a solution of wild-type streptavidin (37.8 µM of tetramer) in buffer (10 mM Tris, 100 mM NaCl, pH 7.4, 25 ºC) was added probe **3** (37.8 µM, from 1 mM stock solution in DMSO) and the resulting solution was gently shaken at 4 °C for 2 h. Then, the next biotinylated ligand (biotin-X-DHPE **9**, biotin **11**, desthiobiotin **12**, or probe **3**) was added (113.4 µM) and the solution was shaken again at 4 °C for 2 h. The resulting stock solution was used as such for the fluorescence spectroscopy and imaging experiments without further purification.

4. Preparation of LUVs

DOPC LUVs. A thin lipid film was prepared by evaporating a solution of DOPC (30 µmol) in $CHCl_3$ (1 mL) on a rotary evaporator (rt) and then under vacuum overnight. The resulting film was

hydrated with a buffer (1.0 mL, 10 mM Tris, 100 mM NaCl, pH 7.4) for 30 min at rt, subjected to freeze-thaw cycles (5×, liquid N_2, 55 °C water bath) and extruded (15×) through a polycarbonate membrane (pore size, 100 nm) using Mini-extruder. Thus obtained DOPC LUVs stock dispersion had following characteristics, and was used for the spectroscopic measurements without further purifications: 30 mM DOPC in 10 mM Tris, 100 mM NaCl, pH 7.4.

DPPC LUVs were prepared similarly using DPPC instead of DOPC. Hydration and extrusion were performed at 55 ºC. DPPC LUVs stock: 30 mM DPPC in 10 mM Tris, 100 mM NaCl, pH 7.4.

SM/CL LUVs. A thin lipid film was prepared by evaporating a 7:3 solution of SM/CL (10 µmol) in MeOH/$CHCl_3$ 1:1 (1 mL) on a rotary evaporator (rt) and then under vacuum overnight. The resulting film was hydrated with a buffer (1.0 mL, 10 mM Tris, 100 mM NaCl, pH 7.4) for 30 min at 55 ºC with occasional bath sonication. The resulting dispersion was subjected to freeze-thaw cycles (5×, liquid N_2, 55 °C water bath) and extruded (15×) through a polycarbonate membrane (pore size, 100 nm) at 50 ºC. Obtained SM/CL LUVs stock dispersion had following characteristics and was used for the spectroscopic measurements without further purifications: 10 mM SM/CL in 10 mM Tris, 100 mM NaCl, pH 7.4.

DOPC/biotin-X DHPE LUVs. A thin lipid film was prepared by evaporating a 100-*x*:*x* (molar ratio) DOPC/biotin-H DPHE (10 µmol) solution in $CHCl_3$ (1 mL) under a gentle nitrogen flux. The resulting film was hydrated with a buffer (1.0 mL, 10 mM Tris, 100 mM NaCl, pH 7.4) for 30 min at rt, subjected to freeze-thaw cycles (5×, liquid N_2, 55 °C water bath) and extruded (15×) through a polycarbonate membrane (pore size, 100 nm) using Mini-extruder. Thus obtained DOPC/biotin-X DHPE(*x*%) LUVs stock dispersion had following characteristics, and was used for the spectroscopic measurements without further purifications: 10 mM DOPC/biotin-X DHPE (100-*x*:*x*) in 10 mM Tris, 100 mM NaCl, pH 7.4.

DPPC/biotin-X DHPE LUVs were prepared similarly using DPPC instead of DOPC. Hydration and extrusion were performed at 55 ºC. DPPC LUVs stock: 10 mM DPPC/biotin-X DHPE (100-*x*:*x*) in 10 mM Tris, 100 mM NaCl, pH 7.4.

SM/CL/biotin-X DHPE LUVs. A thin lipid film was prepared by evaporating a 70-0.5*x*:30-0.5*x*:*x* solution of SM/CL/biotin-X DHPE (10 µmol) in MeOH/$CHCl_3$ 1:1 on a rotary evaporator (rt) and then under vacuum overnight. The resulting film was hydrated with a buffer (1.0 mL, 10 mM Tris, 100 mM NaCl, pH 7.4) for 30 min at 50 ºC with occasional bath sonication. The resulting dispersion was subjected to freeze-thaw cycles (5×, liquid N_2, 55 °C water bath) and extruded (15×) through a polycarbonate membrane (pore size, 100 nm) at 55 ºC. Obtained SM/CL LUVs stock dispersion had following characteristics and was used for the spectroscopic measurements without further purifications: 10 mM SM/CL/biotin-X DHPE (70-0.5*x*:30-0.5*x*:*x*) in 10 mM Tris, 100 mM NaCl, pH 7.4.

5. Fluorescence measurements in LUVs

Standard fluorescence measurements. To a gently stirred buffer solution (2.0 mL, 10 mM Tris, 100 mM NaCl, pH 7.4, 25 ºC) in a quartz cuvette were added LUVs (75 µM final concentration, 5 µL of 30 mM or 15 µL of 10 mM of stock solutions previously prepared). Emission (λ_{ex} = 430 nm) and excitation spectra (λ_{em} = 570 nm) of the dispersion were recorded to serve as backgrounds. The probe **3** (0.5 µL of 1 mM stock solution in DMSO) or streptavidin complexes (13.2 µL of 37.8 µM stock solution in buffer) were added to reach the desired final concentration (here 250 nM) and the solutions were gently stirred for 15 min at rt before spectra measurement. Obtained spectra were background subtracted and corrected using the correction function supplied by the manufacturer.

Insulin complexation experiments. Complex **26** (1 µM, 53 µL of a 37.8 µM stock solution in buffer solution) was attached to DPPC vesicles containing 5% of lipid **9** and was measured as described above. Then, biotinylated insulin (1 mM solution in buffer solution) was added in different amounts (1 equiv.: 2 µL, 2 equiv.: 4 µL; 10 equiv.: 20 µL). The resulting solution was gently stirred for 5 min at rt before spectra measurement. Obtained spectra were background subtracted and corrected using the correction function supplied by the manufacturer.

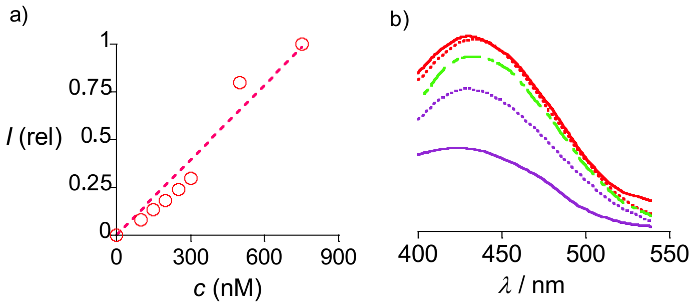

Fig. S1 a) Dependence of fluorescence intensity on the concentration of complex **26** fifteen minutes after addition to L_d DOPC LUVs containing 5% biotin-X DHPE **9**. b) Excitation spectra of complex **26** (0.25 µM) recorded fifteen minutes after addition to DOPC LUVs with 0 (purple, solid), 2 (purple, dotted), 5 (red, dotted), 10 (red, solid) and 20 mol% biotin-X DHPE **9** (green, dashed).

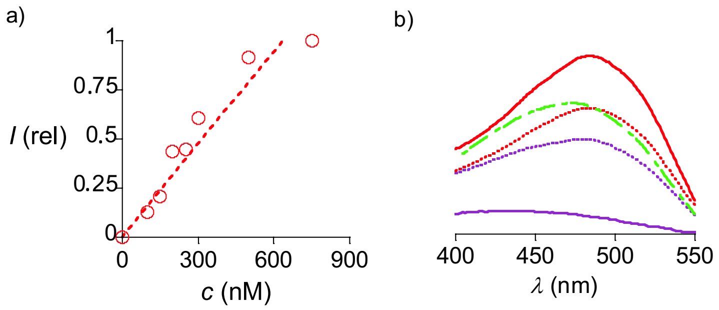

Fig. S2 a) Dependence of fluorescence intensity on the concentration of complex **26** fifteen minutes after addition to S_o DPPC LUVs containing 5% biotin-X DHPE **9**. b) Excitation spectra of complex **26**

(0.25 μM) recorded fifteen minutes after addition to DPPC LUVs with 0 (purple, solid), 2 (purple, dotted), 5 (red, dotted), 10 (red, solid) and 20 mol% biotin-X DHPE **9** (green, dashed).

6. Preparation of GUVs

GUVs were prepared by electroformation method using a Nanion Vesicles Prep Pro device made of two ITO electrodes.

DOPC GUVs. A thin lipid film was prepared by evaporating a solution of DOPC (10 mM) in $CHCl_3$ (20 μL) on the conductive side of an ITO electrode and further drying under vacuum overnight. Then, an o-ring covered in silicon grease was placed around the film, and 250 μL of an aqueous sucrose solution (250 mM) was added onto the film. The second ITO electrode was placed on top of the first one, conductive side toward the joint and the sucrose solution. The electrodes were placed in the electroformation device and were exposed to an electric field of 1.2 V and 10 Hz for 5 h at 25 °C. This resulting stock solution of GUVs was used without further purification, after eventual dilution in 10 mM Tris, 100 mM NaCl, pH 7.4 for the microscopy experiments.

DOPC/biotin-X-DHPE(5%) GUVs. A procedure identical to DOPC GUVs was followed, using a 95:5 solution of DOPC/biotin-X-DHPE (10 mmol) in $CHCl_3$ (20 μL). The resulting stock solution of GUVs was used without further purification, after eventual dilution in 10 mM Tris, 100 mM NaCl, pH 7.4 for the microscopy experiments.

SM/CL GUVs. A procedure identical to DOPC GUVs was followed, using a 1:1 solution of SM/CL (10 mmol) in chloroform (20 μL). The drying of the lipid film and the electroformation were performed at 55 °C. The resulting stock solution of GUVs was used without further purification, after dilution in 10 mM Tris, 100 mM NaCl, pH 7.4 for the microscopy experiments.

SM/CL(rhod) GUVs. A procedure identical to SM/CL GUVs was followed, using sulforhodamine 101 (0.5 μM) in sucrose solution (250 mM) for the eletroformation. The resulting stock solution of GUVs containing the encapsulated dye was used without further purification, after dilution in 10 mM Tris, 100 mM NaCl, pH 7.4 for the microscopy experiments.

SM/CL/biotin-X-DHPE(5%) GUVs. A procedure identical to DOPC GUVs was followed, using a 47.5:47.5:5 solution of SM/CL/biotin-X-DHPE (10 mmol) in $CHCl_3$ (20 μL). The drying of the lipid film and the electroformation were performed at 55 °C. The resulting stock solution of GUVs was used without further purification, after dilution in 10 mM Tris, 100 mM NaCl, pH 7.4 for the microscopy experiments.

7. Studies in GUVs

Confocal laser scanning microscopy. GUVs stock solutions resulting from the procedure reported above were diluted (10 µL in 190 µL) before being deposed on a 35 mm glass bottom dish. The probe was added in order to reach the desired concentration and the liquid was very carefully mixed with a pipette. For the experiments involving the biotinylated probe **3** or any streptavidin complex, an argon laser (λ_{ex} = 488 nm, 30% laser power) was used and the fluorescence was collected between 550 and 650 nm. For dual dye experiments, images were obtained from sequential scans using a white laser at 70% laser power (λ_{ex1} = 488 nm, collection between 550 and 650 nm, 50% laser power, and λ_{ex2} = 584 nm, collection between 600 and 700 nm, 20% laser power).

Fluorescence lifetime imaging microscopy. Samples were prepared as for CLSM imaging. Laser at 485 nm (PicoQuant, LDH-D-C-485) was used and the fluorescence was collected between 550 and 650 nm. FLIM images were analyzed using SymPhoTime 64 software from PicoQuant.

8. Studies in HeLa cells

The cells were grown 72 h in DMEM in 35 mm glass bottom dishes. Then, the medium was removed and the cells were washed with PBS and twice with Leibovitz's medium. Leibovitz's medium containing the dye at the desired concentration was then added to the cells before imaging. Similar procedure was followed for biotinylated HeLa cells, with the difference of the presence of 25 µg/mL of DSPE-PEG(2000) biotin in the DMEM. For the CLSM, an argon laser (λ_{ex} = 488 nm, 30% laser power) was used and the fluorescence was collected between 550 and 650 nm. For FLIM, a laser at 485 nm (PicoQuant, LDH-D-C-485) was used and the fluorescence was collected between 550 and 650 nm. FLIM images were analyzed using SymPhoTime 64 software from PicoQuant.

Hypertonic osmotic shock. The cells were imaged in 2 mL of Leibovitz's media. Then, 1 mL of media was removed using a 1 mL micropipette and replaced by 1 mL of 1 M sucrose solution. The cells were imaged again right after the shock.

9. NMR spectra

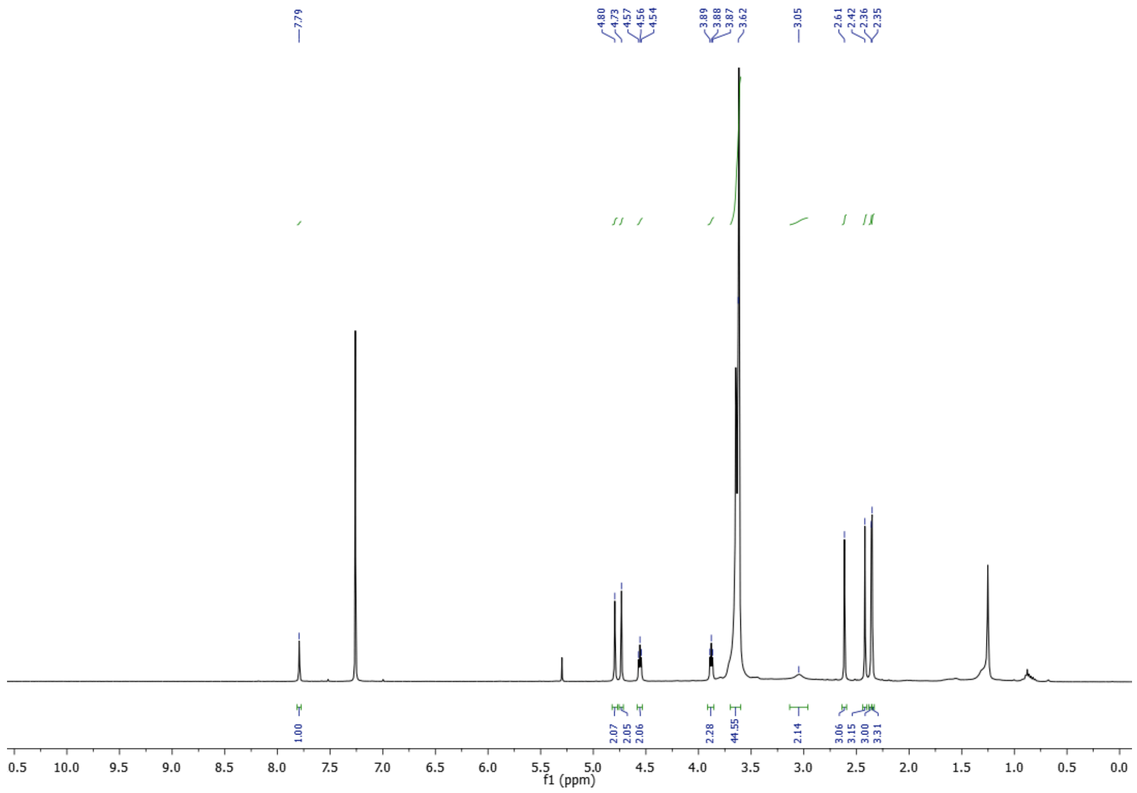

Fig. S3 ^{1}H NMR spectrum of compound **44** in $CDCl_3$.

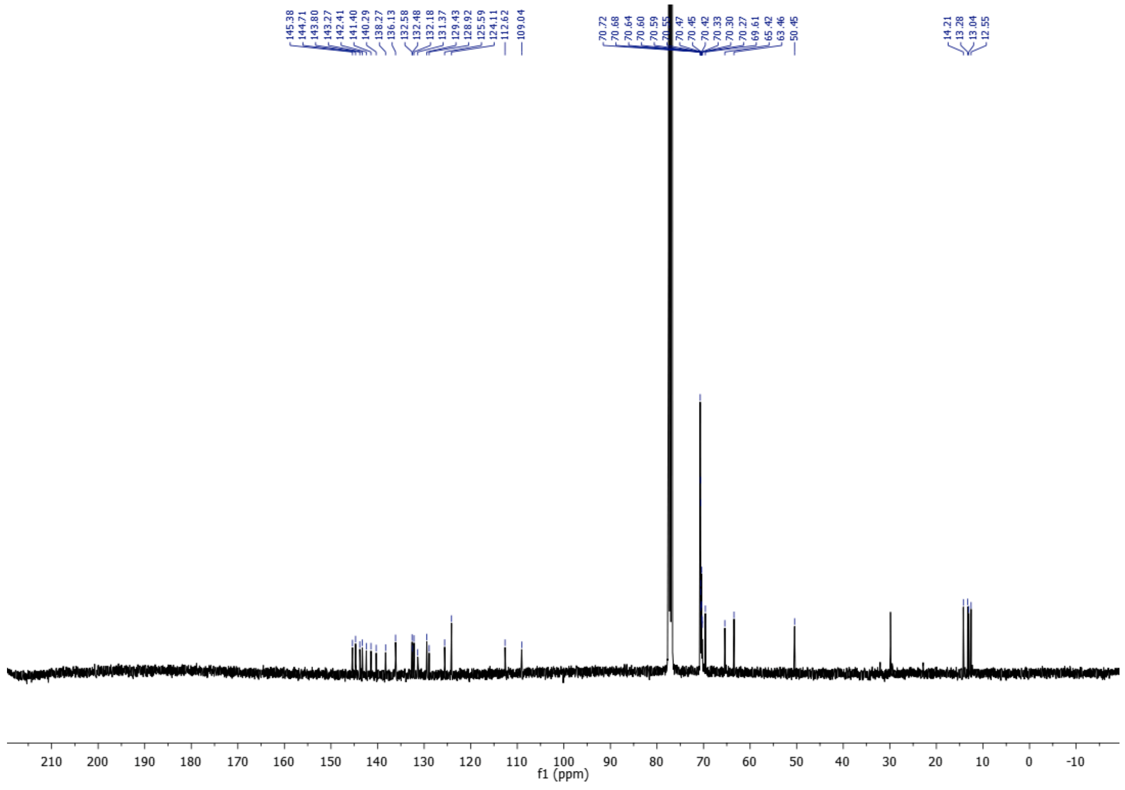

Fig. S4 ^{13}C NMR spectrum of compound **44** in $CDCl_3$.

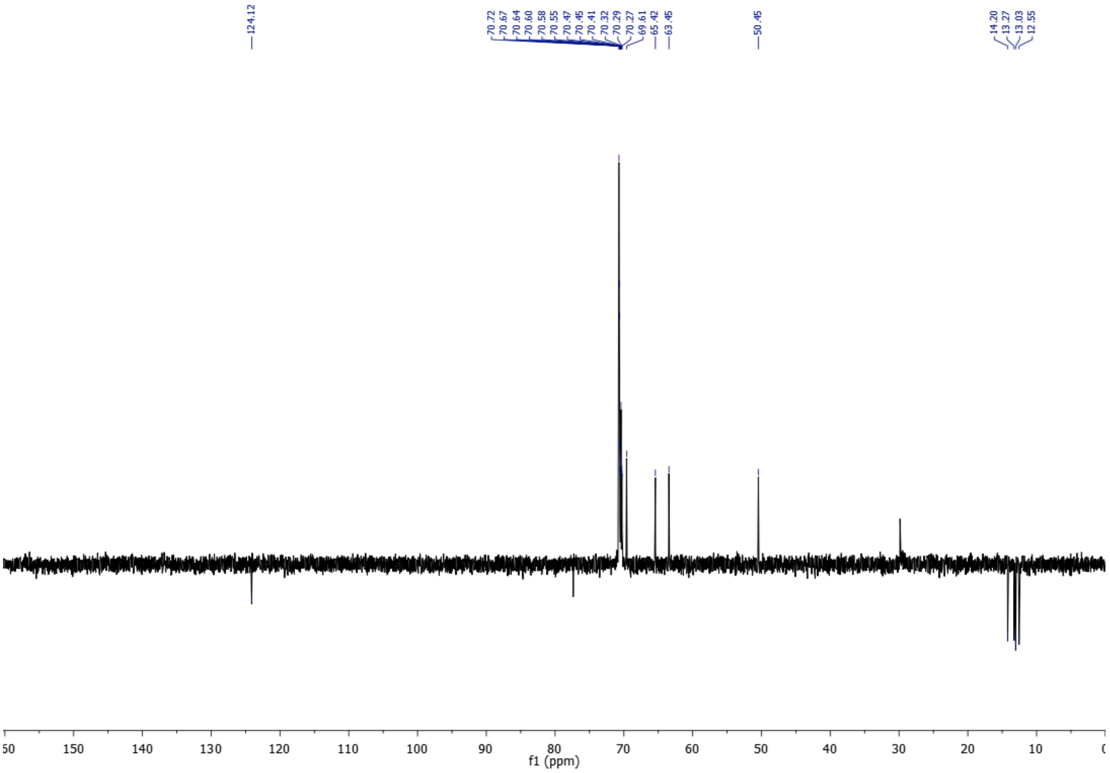

Fig. S5 DEPT-135 ^{13}C NMR spectrum of compound **44** in $CDCl_3$.

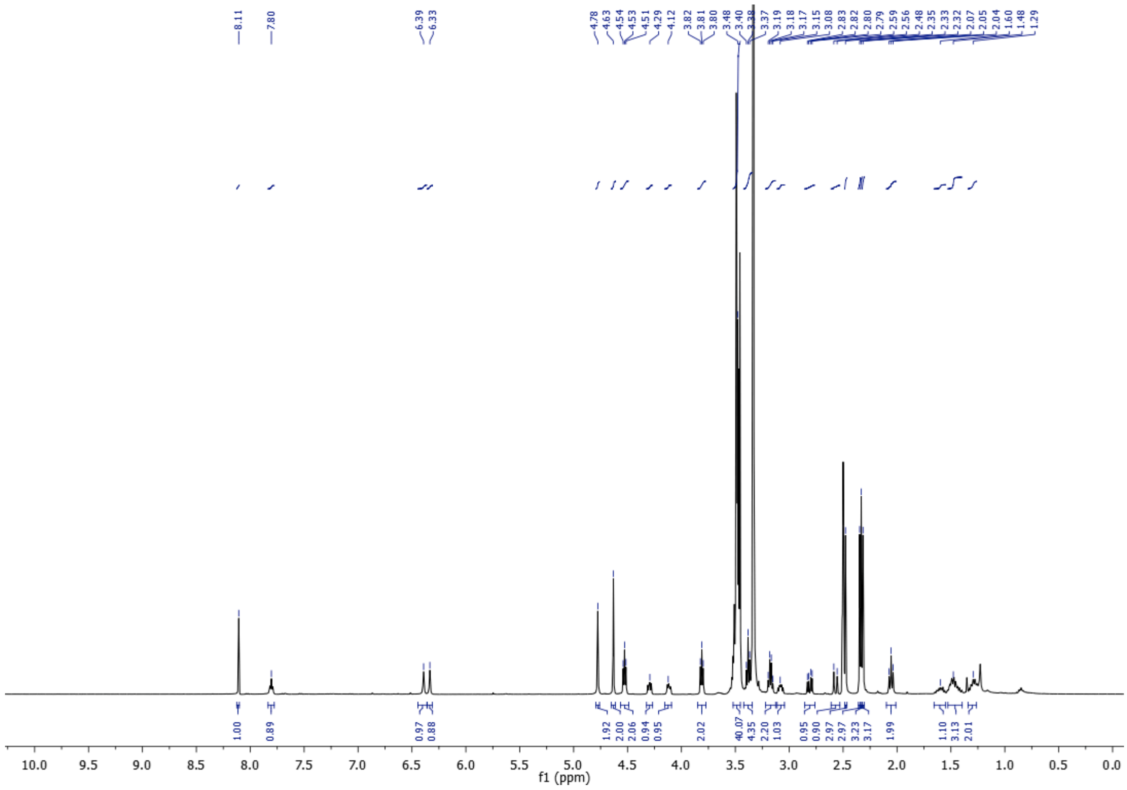

Fig. S6 ^{1}H NMR spectrum of compound **3** in DMSO-d_6.

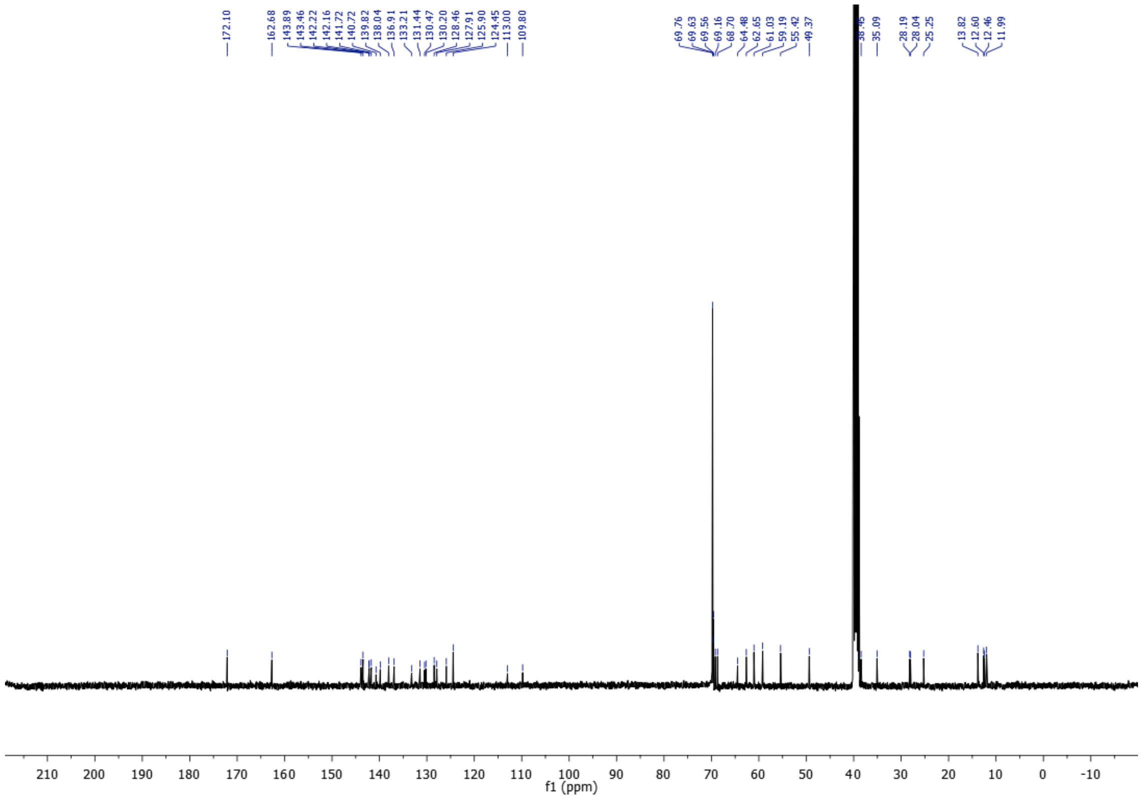

Fig. S7 ^{13}C NMR spectrum of compound **3** in DMSO-d_6.

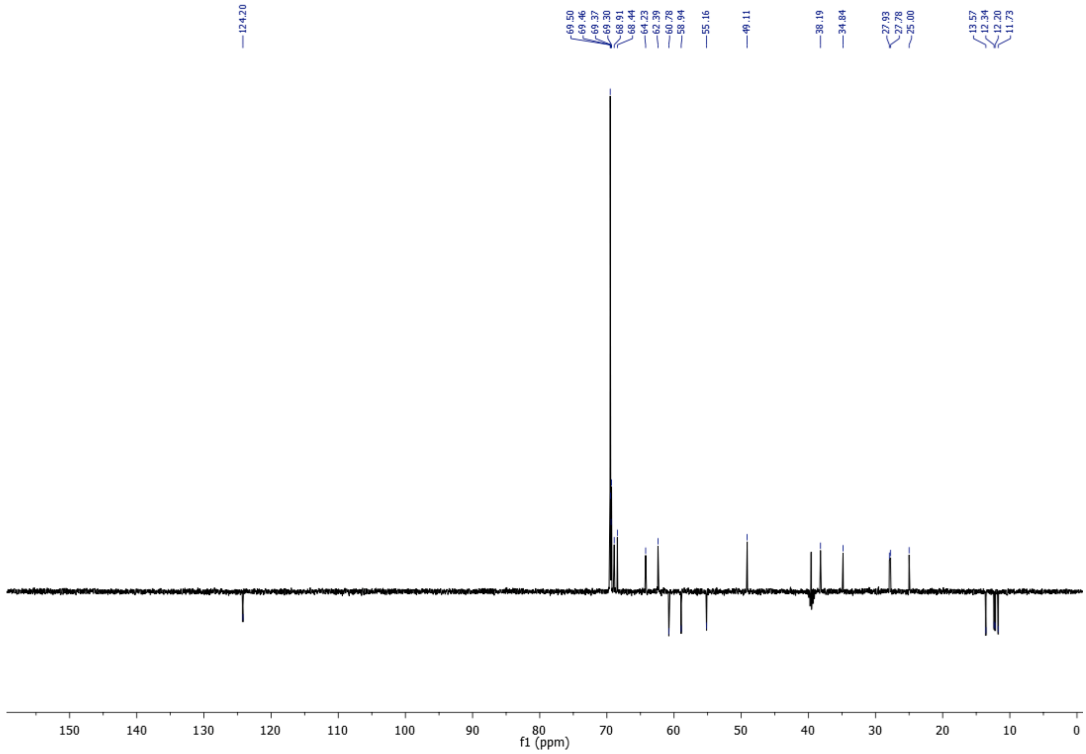

Fig. S8 DEPT-135 ^{13}C NMR spectrum of compound **3** in DMSO-d_6.

10. Supporting references

(S1) S. Soleimanpour, A. Colom, E. Derivery, M. Gonzalez-Gaitan, A. Roux, N. Sakai and S. Matile, *Chem. Commun.*, 2016, **52**, 14450–14453.

(S2) A. Doerflinger, N. N. Quang, E. Gravel, G. Pinna, M. Vandamme, F. Dé, R. Ducongé and E. Doris, *Chem. Commun.*, 2018, **54**, 3613–3616.
